# N-acetylcysteine can be the key to tackling substance use disorders

**DOI:** 10.1192/j.eurpsy.2021.1540

**Published:** 2021-08-13

**Authors:** M. Alves, L. Paulino Ferreira, D. Durães, A. Gamito

**Affiliations:** Psychiatry Department, Hospital Center of Setúbal, Setúbal, Portugal

**Keywords:** N-acetylcysteine, psychiatry, Substance Use Disorder

## Abstract

**Introduction:**

N-acetylcysteine (NAC) is a precursor of cysteine and glutathione, widely known as an antidote to paracetamol overdose. Its role as precursor of an antioxidant and modulating agent of glutamatergic, dopaminergic, neurotropic and inflammatory pathways, raised interest in its application in psychiatric disorders. NAC emerges as a promising therapeutic agent in substance use disorders (SUD) and provides a treatment option in a field with limited and suboptimal therapies.

**Objectives:**

To describe the use of NAC in SUD (tobacco, cocaine, cannabis, methamphetamine and alcohol), its potential mechanisms and clinical application.

**Methods:**

The literature was searched using the Pubmed database with the following keywords “N-acetylcysteine”, “Substance use disorders” and “Psychiatry”. Retrieved papers (2011-2018) were selected according to their relevance.

**Results:**

SUD results in disruption of glutamate system, in nucleus accumbens, a critical brain area in the rewarding system. NAC reestablishes glutamate homeostasis restoring function of the cysteine-glutamate exchange in glial cells and reversing the downregulated GLT-1 receptor. Concerning its properties, evidence suggests that NAC is able to decrease drive, craving or compulsion to consume, making it particular useful in relapse prevention after achieving abstinence.

**Conclusions:**

NAC has revealed itself as a promising therapeutic agent in SUD and its safety profile and favourable tolerability, as well as being an over-the-counter medication, adds to its interest. Data is still preliminary for the use of NAC in psychiatry disorders, due to the relatively small number of trials and their heterogeneous methodology. Larger studies are needed to confirm efficacy, optimal doses, long-term tolerability and side effects.

